# A novel nomogram predicting overt hepatic encephalopathy after transjugular intrahepatic portosystemic shunt in portal hypertension patients

**DOI:** 10.1038/s41598-023-42061-w

**Published:** 2023-09-14

**Authors:** Yong Liao, Lin Zhang, Ji-tao Wang, Zhen-dong Yue, Zhen-hua Fan, Yi-fan Wu, Yu Zhang, Cheng-bin Dong, Xiu-qi Wang, Ting Cui, Ming-ming Meng, Li Bao, Shu-bo Chen, Fu-quan Liu, Lei Wang

**Affiliations:** 1 Department of Interventional Therapy, Beijing Shijitan Hospital, Capital Medical University, No. 10 Tie Yi Road, Yangfangdian, Haidian District, Beijing, 100038 China; 2 Department of Gastroenterology, Beijing Shijitan Hospital, Capital Medical University, Beijing, 100038 People’s Republic of China; 3 Department of Pharmacy, Beijing Shijitan Hospital, Capital Medical University, Beijing, 100038 People’s Republic of China; 4 Department of Hepatobiliary Interventional Therapy, Beijing Tsinghua Changgung Hospital, Beijing, China; 5 School of Clinical Medicine, Tsinghua University, Beijing, 100038 People’s Republic of China; 6 Department of Hepatobiliary Surgery, Xingtai People’s Hospital of Hebei Medical University, Xingtai, 054001 Hebei People’s Republic of China

**Keywords:** Gastroenterology, Medical research

## Abstract

We aim to develop a nomogram to predict overt hepatic encephalopathy (OHE) after transjugular intrahepatic portosystemic shunt (TIPS) in patients with portal hypertension, according to demographic/clinical indicators such as age, creatinine, blood ammonia, indocyanine green retention rate at 15 min (ICG-R15) and percentage of Portal pressure gradient (PPG) decline. In this retrospective study, 296 patients with portal hypertension who received elective TIPS in Beijing Shijitan Hospital from June 2018 to June 2020 were included. These patients were randomly divided into a training cohort (n = 207) and a validation cohort (n = 89). According to the occurrence of OHE, patients were assigned to OHE group and non-OHE group. Both univariate and multivariate analyses were performed to determine independent variables for predicting OHE after TIPS. Accordingly, receiver operating characteristic (ROC) curve, calibration curve, and decision curve analysis (DCA) were used to compare the accuracy and superiority of a novel model with conventional Child–Pugh and MELD scoring model. Age (OR 1.036, 95% CI 1.002–1.070, p = 0.037), Creatinine (OR 1.011, 95% CI 1.003–1.019, p = 0.009), Blood ammonia (OR 1.025, 95% CI 1.006–1.044, p = 0.011), ICG-R15 (OR 1.030, 95% CI 1.009–1.052, p = 0.004) and Percentage decline in PPG (OR 1.068, 95% CI 1.029–1.109, p = 0.001) were independent risk factors for OHE after TIPS using multifactorial analysis. A nomogram was constructed using a well-fit calibration curve for each of these five covariates. When compared to Child–Pugh and MELD score, this new nomogram has a better predictive value (C-index = 0.828, 95% CI 0.761–0.896). Consistently, this finding was reproduceable in validation cohort and confirmed with DCA. A unique nomogram was developed to predict OHE after TIPS in patients with PHT, with a high prediction sensitivity and specificity performance than commonly applied scoring systems.

## Introduction

After a long-term inflammatory response, the normal liver parenchyma is replaced by fibrotic tissues and regenerative nodules, which can subsequently progress to cirrhosis, and eventually lead to portal hypertension (PHT)^1^. The PHT can cause common clinical manifestations such as rupture and bleeding of esophageal and gastric varices, and even intractable ascites^2^, making it difficult to treat. In recent years, transjugular intrahepatic portosystemic shunt (TIPS) has been widely used in PHT patients. With well-accepted advantages of minimally invasive, definite curative effects, and quick postoperative recovery, TIPS has become one of the main options for PHT treatment^3^. However, there are many complications after TIPS. For example, hepatic encephalopathy (HE) is a common postoperative complication after TIPS, which seriously affects the prognosis of patients and significantly increases the postoperative mortality risk^4–7^.

According to clinical manifestations, HE can be divided into covert hepatic encephalopathy (CHE) and overt hepatic encephalopathy (OHE). In addition, HE may be categorized into grades 0 to 4 according to the West Haven grading criteria; of which, CHE includes grades 0 and 1, whereas OHE includes grades 2–4^8^. Previous studies have suggested that the incidence of HE after TIPS can be as high as 5–35%^9–11^. Many risky factors, such as advanced age, Child–Pugh score and HE medical history can predict HE after TIPS in PHT patients^12^. The gradient change in portal venous pressure before and after operation is closely related to HE^13^. In addition, stent diameter and preoperative serum albumin level might be independent risk factors for HE in patients after TIPS^14^.

Presently, Child–Pugh and MELD scoring systems for predicting HE after TIPS are clinically available^15^. Intraoperative events can affect susceptibility to HE after TIPS. Unfortunately, conventional models do not include surgery-related factors, such as stent diameter, puncture site and intraoperative pressure measurement, which cannot be used to accurately predict HE after TIPS. Recently, a prediction model related to OHE after TIPS has been developed^16^. Although great efforts have been made to predict OHE after TIPS, certain limitations exist due to a lack of risk factors and small sample sizes. Therefore, it is necessary to establish a more accurate prediction model for predicting the risk of OHE after TIPS in PHT patients. A novel nomogram prediction model can become an intuitive and convenient tool. Therefore, in this study, a nomogram prediction model that can more intuitively predict OHE after TIPS will be constructed to replace the conventional scoring systems. This model will help to evaluate patients at high risk of OHE and implement preventive and therapeutic measures after TIPS, thereby providing strategies for the treatment of PHT.

## Materials and methods

### Patients

In this retrospective study, 358 PHT patients who received elective TIPS at Beijing Shijitan Hospital from June 2018 to June 2020 were included. Among them, 62 patients were excluded due to incomplete data and loss to follow-up. The remaining 296 patients were randomly divided into a training cohort (n = 207) and a validation cohort (n = 89), All patients with esophagogastric variceal bleeding and refractory ascites had treatment failure after first-line treatment. This study was approved by the Institutional Review Board at Beijing Shijitan Hospital in accordance with the Declaration of Helsinki. All methods are performed in accordance with relevant guidelines and regulations.

The inclusion criteria were as follows: (1) diagnosed with PHT; (2) treatment of varicose bleeding by beta-blockers or endoscopy failed; (3) patients with refractory ascites who do not respond to adequate doses of diuretics and sodium restriction; (4) with complete perioperative clinical data. The exclusion criteria were as follows: (1) diagnosed with preoperative HE; (2) previously treated with liver transplantation; (3) diagnosed with preoperative severe cardiopulmonary insufficiency or severe encephalopathy.

### Clinicopathological variables

Demographic and clinical data were collected, including age, sex, history of PHT and liver diseases. Imaging results from MRI contrast-enhanced, CT contrast-enhanced, and ultrasonography were analyzed to identify cirrhosis and ascites. TIPS perioperative tests were recorded, including complete blood count, renal function, serum electrolyte, plasma ammonia, liver function, prothrombin time, international normalized ratio and ICG-R15. The perioperative information on TIPS was recorded. In addition, the symptoms of OHE were evaluated by more than 2 experienced hepatologists according to the West-Haven criteria.

### Diagnosis and definitions

The MELD score^17^ was calculated as 11.2 × ln(INR) + 9.57 × ln(Cr, mg/dL) + 3.78 × ln(TBIL, mg/dL) + 6.43. Three continuous factors (total bilirubin, albumin, and prothrombin time) and two categorical variables (ascites, hepatic encephalopathy) comprised the Child–Pugh scoring system^18^. According to Child–Pugh scores, patients were categorized into three grades: grade A (5–6 points), grade B (7–9 points), and grade C (10–15 points). The PHT was detected in patients with esophageal varices visible during endoscopy or with a low platelet count (100 × 10^9^/L) related to splenomegaly (a diameter of larger than 12 cm on ultrasonography, CT, or MRI)^19^. After excluding other possible causes of altered mental status, HE was defined as neuropsychiatric abnormalities from mild neuropsychological dysfunction to deep coma and abnormal ammonia levels^20^. The severity/stage of HE was evaluated. All patients were followed up at 1, 3, 6, and 12 months after TIPS, and the onset characteristics of OHE (with West-Haven score ≥ grade 2) were recorded. All OHE occurred within 3 months after TIPS, so post-TIPS OHE was defined as OHE that occurred within 3 months after TIPS. Because CHE has a relatively small effect on quality of life, it is difficult to quantify in long-term follow-up and it is difficult to include CHE in statistical analysis, so only OHE was included in statistical analysis. All patients underwent ICG-R15 testing using a dye density graph analyzer (DDG-3300K, Japan) and ICG clearance test reagent (Japan, NIHON KOHDEN). The plasma ICG concentration was measured 15 min after injection of ICG (0.5 mg/kg).

### PPG measurement method

TIPS was established under local anesthesia using a transjugular approach. 4-Fr pigtail catheters were placed in the right atrium, inferior vena cava (at the level of the hepatic vein), and portal vein (where the spleen and superior mesenteric vein converge) to measure the pressure before and after stent placement. For all measurements, the pressure transducer was calibrated to 0 mmHg at the level of the patient's axillary midline. Each measurement was performed three times and then averaged. PPG is the pressure difference obtained by subtracting the IVC pressure from the PV pressure.

### Statistical analysis

The statistical analysis was performed by SPSS 26.0 (SPSS Inc, Chicago, USA) and R 4.0.3 (Institute of Statistics and Mathematics). The univariate or multivariate analysis was conducted at a test level of 0.01 or 0.05, respectively. Means of continuous variables were calculated and compared using Mann–Whitney U tests or Student's t tests. Statistically significant indicators in the univariate analysis were included in the multivariate analysis. The variables associated with OHE were assessed using logistic regression analyses. The nomogram prediction model was evaluated by establishing consistency index and evaluating calibration curve. We compared the predictive value of this new nomogram model with other predictive OHE scores. In order to determine the accuracy of this nomogram, a calibration chart with 1000 bootstrap samples was applied. DCA was used to measure clinical utility of this nomogram by calculating net benefit at different threshold probabilities.

### Ethical approval

The clinical data were approved by the Ethics Committee of Beijing Shijitan Hospital Affiliated to Capital Medical University. Patients sign informed consent.

## Results

### Clinicopathological characteristics of patients

A total of 296 patients including 203 males (68.58%) and 93 females (31.42%), with an average age of 51.89 years, who met the inclusion criteria were included in this study. All patients were divided into a training cohort and a validation cohort. Of which, OHE occurred in 59 patients (19.93%). These patients were diagnosed with viral hepatitis (n = 179, 60.47%), alcoholic hepatitis (n = 34, 11.49%), cholestatic hepatitis (n = 8, 2.7%), or other liver diseases (n = 75, 25.34%). Among those who underwent TIPS, 188 cases (63.51%) were confirmed with esophageal and gastric variceal bleeding, 60 cases (20.27%) developed intractable ascites, 29 cases (9.8%) suffered from esophageal and gastric variceal bleeding complicated with refractory ascites, and 19 cases (6.42%) had other liver diseases. Totally, 189 cases (63.85%) underwent puncture of left branch of portal vein, 61 cases (20.61%) underwent puncture of right branch of portal vein, while 46 cases (15.54%) underwent the puncture of portal vein bifurcation. The average decrease ratio of portal vein was 38.1% and the average decrease value of portal vein was 10.73 mmHg. The clinicopathological characteristics of these patients were summarized in Table 1.

**Table 1:** Characteristics of patients in training cohort and validation cohort.

Characteristics	Training (n = 207)	Validation (n = 89)	p value
OHE			
No	168 (81.16)	69 (77.53)	0.5262
Yes	39 (18.84)	20 (22.47)	
Age, years	51.46 ± 12.88	52.89 ± 12.64	0.2580
Gender			
Female	62 (29.96)	31 (34.8)	0.4156
Male	145 (70.04)	58 (65.2)	
TIPS indication			
Variceal bleeding	123 (59.4)	65 (73)	0.0900
Ascites	49 (23.7)	11 (12.4)	
Bleeding combine with ascites	20 (9.7)	9 (10.1)	
Other	15 (7.2)	4 (4.5)	
Causes of cirrhotic			
Virus	127 (61.35)	52(58.4)	0.9025
Alcoholic	24 (11.59)	10 (11.2)	
Biliary	6 (2.89)	2 (2.2)	
Other	50 (24.15)	25 (28.1)	
Stent size, mm			
7	42 (20.3)	22 (24.7)	0.3648
8	144 (69.6)	62 (69.7)	
10	21 (10.1)	5 (5.6)	
Puncture site of portal vein			
Left branch	122 (58.9)	67 (75.3)	0.0087
Right branch	52 (25.1)	9 (10.1)	
Bifurcation	33 (15.9)	13 (14.6)	
Child–Pugh grade			
A Class	79 (38.2)	40 (44.9)	0.5512
B Class	110 (53.1)	42 (47.2)	
C Class	18 (8.7)	7 (7.9)	
MELD score	6.42 (3.78–10.16)	6.78 (4.22–9.70)	0.9386
ICG-R15	26.3 (18–45)	33.6 (21.15–52.8)	0.0432
PPG decline in value, mmHg	11 (8–14)	10 (7–14)	0.5631
Percentage decline in PPG	45.45 (40–52.38)	44.44 (33.33–52.28)	0.1823
ALT, U/L	21 (14–32)	19 (13–27)	0.1927
AST, U/L	28 (20–42)	27 (23–37)	0.5304
Albumin, g/L	34.72 ± 5.31	34.99 ± 4.75	0.6552
Total bilirubin, μmol/L	23.7 (16.9–35.6)	23.9 (16.5–32)	0.3466
Creatinine, μmol/L	60 (51–74)	62 (52–72.5)	0.7552
PT, s	14.5 (13.1–16.2)	14.4 (13.2–16.25)	0.8248
INR	1.31 (1.20–1.45)	1.33 (1.21–1.48)	0.4073
BUN, mmol/L	5.53 (4.20–7.71)	5.17 (4.10–7.28)	0.2996
Na, mmol/L	139 (136–142)	140 (136–141)	< 0.001
Blood ammonia, umol/L	45.5 (36.1–58.9)	36.6 (31–48.35)	0.460

*OHE* overt hepatic encephalopathy, *TIPS* transjugular intrahepatic portosystemic shunt, *ALT* alanine transaminase, *AST* aspartate transaminase, *PT* prothrombin time, *MELD* Model For End-Stage Liver Disease, *ICG-R15* indocyanine green retention rate at 15 min, *PPG* Portal pressure gradient, *INR* international normalized ratio.

### Univariate and multivariate analyses of risk factors for OHE after TIPS

In univariate models, age (p = 0.007), Child–Pugh grade (p = 0.001), ICG-R15 (p < 0.001), percentage of PPG decline (p = 0.007), percentage decline in PPG (p < 0.001), creatinine (0.004) and blood ammonia (p = 0.005) were identified as risk factors for OHE after TIPS in the training cohort (Table 2). Then, multivariate logistic analysis was performed on the above potential risk factors. Notably, age (p = 0.037), ICG-R15 (p = 0.004), percentage of PPG decline (p = 0.001), creatinine (0.009), and blood ammonia (p = 0.011) were independent risk factors for OHE after TIPS (Table 2).

**Table 2:** Univariate and multivariate analysis of OHE factors after TIPS.

Characteristics	Univariable logistic regression		Multvariable logistic regression	
	OR (95% CI)	p value	OR (95% CI)	p value
Age, years	1.041 (1.011–1.071)	0.007	1.036 (1.002–1.070)	0.037
Gender, (female vs male)	0.953 (0.448–2.031)	0.902		
TIPS indication	1.141 (0.796–1.637)	0.472		
Causes of cirrhosis	0.889 (0.713–1.107)	0.293		
Stent size, mm, (7 vs 8 vs 10)	0.520 (0.293–0.922)	0.520		
Puncture site of portal vein	1.044 (0.659–1.653)	0.856		
Child–Pugh grade	2.869 (1.576–5.223)	0.001		
MELD score	1.077 (1.017–1.140)	0.011		
ICG-R15	1.032 (1.014–1.051)	< 0.001	1.030 (1.009–1.052)	0.004
Percentage of PPG decline	1.117 (1.031–1.211)	0.007	1.073 (0.932–1.235)	0.329
Percentage decline in PPG	1.064 (1.028–1.101)	< 0.001	1.068 (1.029–1.109)	0.001
ALT, U/L	1.001 (0.997–1.005)	0.545		
AST, U/L	1.004 (0.999–1.009)	0.160		
Albumin, g/L	0.928 (0.869–0.992)	0.027		
Total bilirubin, μmol/L	1.002 (0.995–1.009)	0.568		
Creatinine, μmol/L	1.010 (1.003–1.017)	0.004	1.011 (1.003–1.019)	0.009
PT, s	1.006 (0.871–1.162)	0.937		
INR	0.713 (0.175–2.904)	0.637		
BUN, mmol/L	1.079 (1.016–1.146)	0.013		
Na, mmol/L	0.940 (0.882–1.002)	0.059		
Blood ammonia, umol/L	1.025 (1.007–1.042)	0.005	1.025 (1.006–1.044)	0.011

*TIPS* transjugular intrahepatic portosystemic shunt, *ALT* alanine transaminase, *AST* aspartate transaminase, *PT* prothrombin time, *MELD* Model For End-Stage Liver Disease, *ICG-R15* indocyanine green retention rate at 15 min, *PPG* Portal pressure gradient, *INR* international normalized ratio.

### Constructing a nomogram prediction model for OHE after TIPS

**Figure 1:**
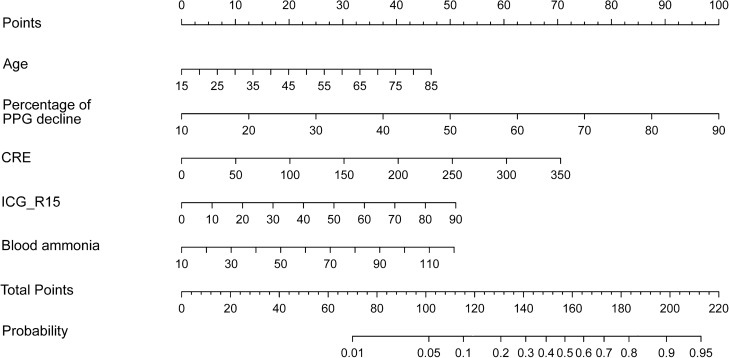
In the training cohort, a novel nomogram was constructed by combining risk variables including age, ICG-R15, percentage of PPG decline, creatinine and blood ammonia.

Based on multivariate analysis, age, ICG-R15, percentage of PPG decline, creatinine, and blood ammonia were identified as independent risk factors for OHE after TIPS. By using rms package of R software, independent factors were incorporated into a nomogram prediction model for OHE after TIPS. The expected probability of OHE after TIPS is obtained by drawing a line to the point axis to obtain the points received by the value of each variable, and the sum of these points corresponds to the total point axis (Fig. 1).

### The nomogram had a higher predictive power for OHE after TIPS in the training cohort

**Figure 2:**
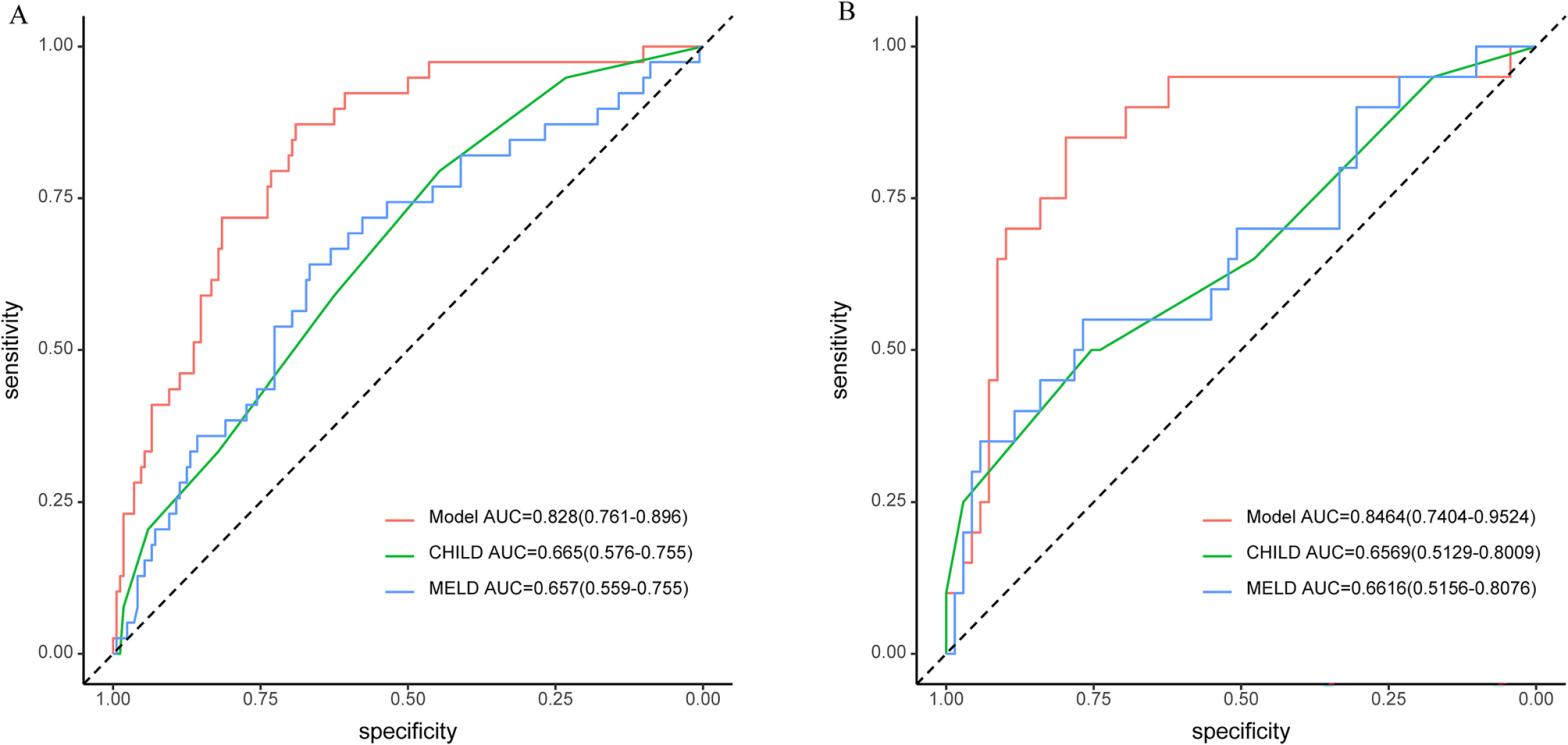
Prediction accuracy of OHE after TIPS was compared with traditional models (Child–Pugh score and MELD score) in (**A**) training cohort and (**B**) validation cohort.

**Figure 3:**
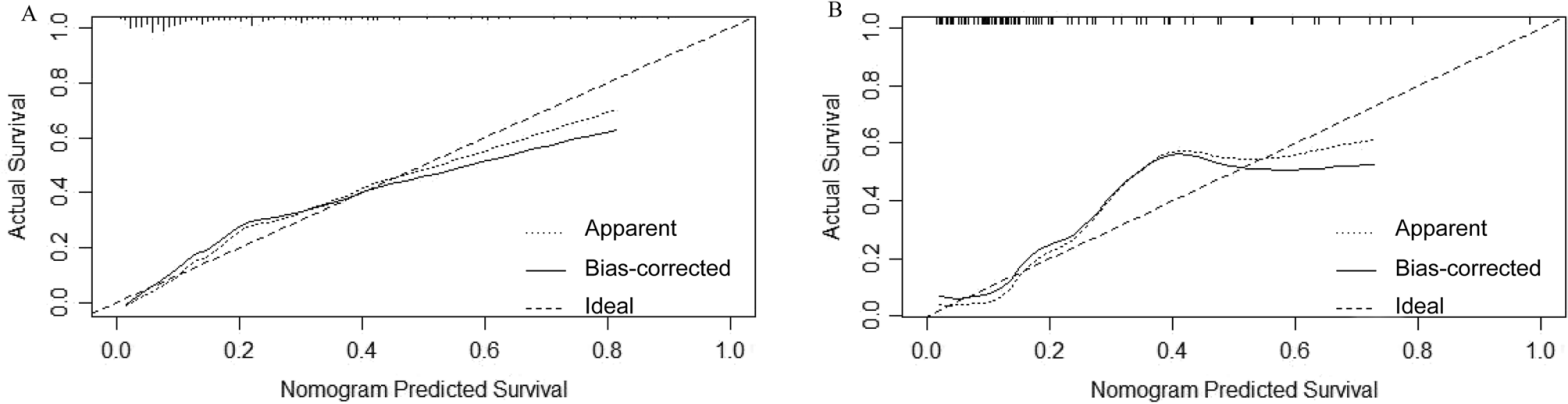
Calibration curves for developing a nomogram in (**A**) training cohort and (**B**) validation cohort. The x-axis depicts expected incidence of OHE after TIPS for this nomogram, whereas the y-axis depicts actual incidence of OHE after TIPS.

In the training cohort, this nomogram model achieved higher prediction accuracy than other conventional models. The C-index of our nomogram was 0.828 (95% CI 0.761–0.896), significantly higher than that of Child–Pugh score (0.665; 95% CI 0.576–0.755) or MELD score (0.657, 95% CI 0.559–0.755) (Fig. 2A). Meanwhile, calibration curve for predicting OHE after TIPS was highly consistent with the observed value. The nomogram-based prediction model could accurately predict OHE after TIPS in the training cohort (Fig. 3A).

### The nomogram had a higher predictive power for OHE after TIPS in the validation cohort

Our nomogram model was accurate in predicting OHE after TIPS in the validation cohort, with a C-index of 0.8464 (95% CI 0.7404–0.9524), higher than that of Child–Pugh score (0.6569, 95% CI 0.5129–0.8009) or MELD score (0.6616, 95% CI 0.5156–0.8076) (Fig. 2B). Meanwhile, calibration curve for predicting OHE after TIPS was highly consistent with the observed value. Therefore, our nomogram-based prediction model could accurately predict OHE after TIPS in the validation cohort (Fig. 3B).

### DCA curve comparison of nomogram and conventional models for OHE after TIPS prediction accuracy

**Figure 4:**
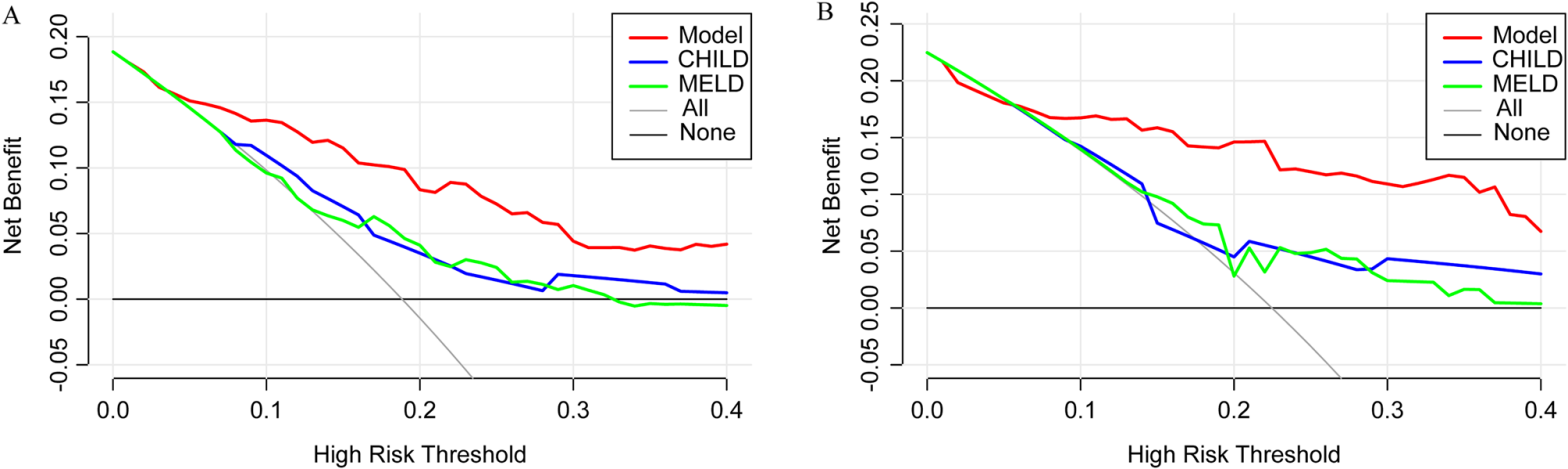
In (**A**) training cohort and (**B**) validation cohort, DCAs were performed on the nomogram and conventional models.

The DCA curves indicated that our nomogram prediction model was more valuable than MELD score or Child–Pugh score (Fig. 4A) in both training and validation cohorts. In validation cohort, this nomogram was more reliable than conventional models (Fig. 4B). The above results indicated that our nomogram was superior to commonly used ones.

## Discussion

With continuous improvement of hardware facilities and technical concepts, effects of TIPS on PHT therapy have becoming more and more remarkable, however, its postoperative complications remain challenging. For example, HE caused by biochemical disturbance of brain functions as a result of liver function injury and portal-systemic shunt greatly affects the quality of life^21,22^. Therefore, implementation of TIPS has been restricted^23^, and it is very important to accurately identify PHT patients who are at risk of OHE after TIPS.

In this study, we have identified that age, creatinine, blood ammonia, ICG-R15, and percentage of PPG decline were independent risk factors for OHE after TIPS in PHT patients based on multivariate logistic regression models. Several risk factors were related to OHE after TIPS in previous studies. For example, age was associated with HE after TIPS^24–27^. For patients who have not experienced HE, preoperative blood ammonia level can be a predictor of HE after TIPS^28^. Similarly, serum creatinine was significantly associated with HE after TIPS^29–31^. The ICG-R15 retention test is a relatively non-invasive method for evaluating liver function, which is used to distinguish patients with acute and chronic liver failure^32^. Presently, it has been widely applied in patients with end-stage liver diseases^33^. Previous studies have suggested that ICG-R15 has clinical value in predicting HE after TIPS^15^ as well as prognosis of patients undergoing hepatectomy^34^. In addition, the more the portal venous pressure decreased after TIPS, the more obvious the effect of portal-systemic shunt was. The risk of postoperative rebleeding was reduced, but the risk of postoperative HE was increased^13^. At present, there are few studies on the percentage of PPG decline in relation to OHE after TIPS. This study has confirmed that the percentage of PPG decline is an independent risk factor for developing OHE after TIPS in PHT patients. At present, the mechanism is not clear. At the same time of the decrease of portal vein pressure, the portal vein blood flow is diverted to the systemic circulation, leading to the reduction of liver blood supply, leading to liver insufficiency and increasing the risk of postoperative HE. In addition, a large amount of ammonia-rich portal blood flow from the gastrointestinal tract directly enters the systemic circulation through the stent, causing an increase in blood ammonia and leading to the occurrence of postoperative HE.

Currently, Child–Pugh scoring system and MELD scoring system have certain predictive value in predicting OHE after TIPS. However, some indicators in Child–Pugh score are subjective or interrelated, so Child–Pugh score cannot accurately predict OHE after TIPS. The MELD score may predict the mortality of patients after TIPS. Presently, MELD is the most widely applied index for evaluating liver function before liver transplantation^35^, and can be helpful to evaluate liver reserve function. Either Child–Pugh score or MELD score lacks indicators that can predict liver function during the operation. Therefore, there are limitations of these conventional models.

The nomogram is an intuitive statistical tool that can provide information for decision-making in clinical practice. Therefore, we combined 5 risk factors to illustrate a combinatory value in predicting OHE after TIPS. Compared with Child–Pugh score and MELD score, our novel model had a better predictive value for OHE after TIPS. The nomogram prediction model, Child–Pugh score and MELD ROC curves were drawn to calculate C-index values. The predictive ability of our nomogram model vs. conventional models in predicting OHE after TIPS was compared. The C-index value of our novel prediction model was the highest in training cohort and validation cohort, indicating that this nomogram prediction model had a better predictive capability. In terms of validation, Bootstrap self-sampling method was used to calculate consistency index. This prediction model had good accuracy. The calibration curve fit well with the ideal curve, indicating that this nomogram prediction model had a good prediction capability for OHE after TIPS. Traditionally, nomograms have been assessed by diagnostic performance indices, and its clinical value cannot be determined. DCA can assess the benefits of various patient-preferred diagnostic tests to identify the risks of undertreatment and overtreatment, in order to facilitate decision-making. The DCA demonstrated that our nomograms provided more benefits than other models in both training and validation cohorts. Therefore, our nomograms can be applied in clinical practice. However, this study still has some limitations: (1) this study used the change value of PPG as one of the model parameters, so the model cannot predict the occurrence of OHE before TIPS; (2) as a retrospective study, there is a certain selection bias; (3) this study only carried out internal verification, but lacked external verification.

In conclusion, we have demonstrated that age, creatinine, ammonia, ICG-R15, and percentage of PPG decline are independent risk factors for predicting OHE after TIPS. When TIPS is performed for PHT patients, in order to reduce the incidence of postoperative OHE, in addition to routine preoperative assessment, attentions should be paid to age and renal function. Furthermore, blood ammonia and ICG-R15 results should be assessed before the surgery. Therapeutic effects attributed to decreased portal venous pressure and the possible increased risk of HE should be balanced during the surgery. In this study, we have proposed a novel predictive nomogram for HE after TIPS. The nomogram harbors good predictive performance and would be a conventional tool to facilitate clinical decision-making.

## Data Availability

The data that support the findings of this study are available on request from the corresponding author. The data are not publicly available due to privacy or ethical restrictions.

## Abbreviations

OHE: Overt hepatic encephalopathy
TIPS: Transjugular intrahepatic portosystemic shunt
ICG-R15: Indocyanine green retention rate at 15 min
PPG: Portal pressure gradient
CI: Confidence interval
DCA: Decision curve analysis
INR: International normalized ratio
CTP: ChildTurcottePugh
MELD: Model for end-stage liver disease
OR: Odds ratio
